# Reproduction of bacterial chemotaxis by a non-living self-propelled object

**DOI:** 10.1038/s41598-023-34788-3

**Published:** 2023-05-20

**Authors:** Yuko Hamano, Kota Ikeda, Kenta Odagiri, Nobuhiko J. Suematsu

**Affiliations:** 1 School of Interdisciplinary Mathematical Sciences, Meiji University, Tokyo, Japan; 2 Graduate School of Advanced Mathematical Sciences, Meiji University, Tokyo, Japan; 3 Meiji Institute for Advanced Study of Mathematical Sciences (MIMS), Meiji University, Tokyo, Japan; 4 School of Network and Information, Senshu University, Kanagawa, Japan

**Keywords:** Biophysics, Biological physics, Chemical physics, Statistical physics, thermodynamics and nonlinear dynamics

## Abstract

Taxic behavior as a response to an external stimulus is a fundamental function of living organisms. Some bacteria successfully implement chemotaxis without directly controlling the direction of movement. They periodically alternate between run and tumble, i.e., straight movement and change in direction, respectively. They tune their running period depending on the concentration gradient of attractants around them. Consequently, they respond to a gentle concentration gradient stochastically, which is called “bacterial chemotaxis.” In this study, such a stochastic response was reproduced by a non-living self-propelled object. We used a phenanthroline disk floating on an aqueous solution of Fe$^{2+}$. The disk spontaneously alternated between rapid motion and rest, similar to the run-and-tumble motion of bacteria. The movement direction of the disk was isotropic independent of the concentration gradient. However, the existing probability of the self-propelled object was higher at the low-concentration region, where the run length was longer. To explain the mechanism underlying this phenomenon, we proposed a simple mathematical model that considers random walkers whose run length depends on the local concentration and direction of movement against the gradient. Our model adopts deterministic functions to reproduce the both effects, which is instead of stochastic tuning the period of operation used in the previous reports. This allows us to analyze the proposed model mathematically, which indicated that our model reproduces both positive and negative chemotaxis depending on the competition between the local concentration effect and it’s gradient effect. Owing to the newly introduced directional bias, the experimental observations were reproduced numerically and analytically. The results indicate that the directional bias response to the concentration gradient is an essential parameter for determining bacterial chemotaxis. This rule might be universal for the stochastic response of self-propelled particles in living and non-living systems.

## Chemotaxis in bacteria and non-living self-propelled objects

**Figure 1:**
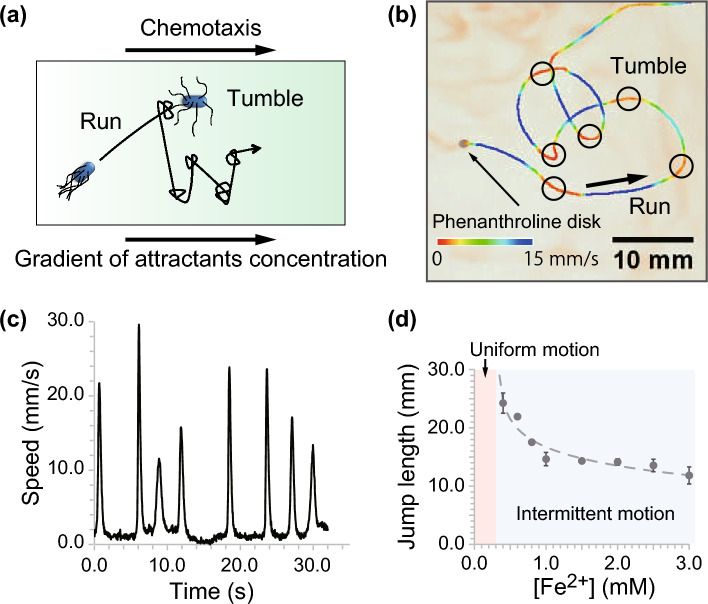
(**a**) Schematic illustration of bacterial motion. The run period of bacteria depends on the concentration of attractants, resulting in gathering in a suitable region, even though the movement direction is isotropic. (**b**) Run-and-tumble motion in the phenanthroline disk. The solid line indicates the trajectory of disk motion. The line color corresponds to the movement speed, as indicated by the color bar at the bottom. (**c**) Speed profile of disk motion. The chemical reaction of phenanthroline and Fe$^{2+}$ prevents disk motion (tumble). The disk then suddenly moves shortly after the depletion of Fe$^{2+}$ around the disk. (**d**) Jump length depending on [Fe$^{2+}$]. The broken line is the fitting curve obtained by Eq. (1).

Bacteria respond to environmental chemical concentration gradients and tend to swim toward regions with suitable conditions^1–3^. However, the size of bacteria is too small to detect differences in the concentration around their bodies^4^. This paradox has been explained using statistical theory. Based on this theory, chemotaxis can be observed stochastically even if the swimming direction of bacteria is isotropic^5–8^. These theoretical approaches were constructed based on the experimentally observed characteristics of bacteria^9–11^. Bacteria show periodic “run” and “tumble” motions (Fig. 1a). In other words, they alternate between ballistic motion (run) and direction change (tumble). Bacteria tune the run period depending on temporal change in the chemical concentration around them during the run, which produces an upward or downward gradient. This leads to their stochastic gathering at regions with suitable conditions.

Non-living self-propelled objects also show chemotaxis, however, they directly change their direction of motion in response to the environmental gradient in most cases^12–15^. This behavior differs significantly from that of bacteria and may not work well if the body size decreases due to the fluctuation effect. Therefore, a bacterial chemotaxis is a crucial strategy for smaller system sizes even for non-living self-propelled objects. Although not run-and-tumble, Sen and Velegol et al. reported an example of such chemotaxis wherein micro-sized metal particles could tune their movement speed depending on the concentration of H$_2$O$_2$, resulting in particle accumulation at high-concentration regions^16^. This can be understood by the mechanism of speed-dependent chemotaxis^17^.

In this study, we demonstrated bacterial chemotaxis using macroscopic self-propelled objects, in which the movement was determined by the iron ion concentration around the objects. It is known that the objects show alternating run-and-tumble motion similar to bacteria^18^. To the best of our knowledge, this is the first study to demonstrate chemotaxis based on the characteristic run-and-tumble motion of self-propelled objects. In addition, we propose a simple agent-based model to reproduce our observations and obtain the distribution function of the objects through a mathematical analysis of the model.

## Run-and-tumble motion of non-living self-propelled objects

Self-propelled objects can exhibit “run-and-tumble” motion coupled with a chemical reaction^19^. Examples of such objects include a phenanthroline solid disk coupled with a complex reaction^18^, a benzoquinone solid disk coupled with a redox reaction^20^, a camphoric-acid solid disk coupled with a neutralization reaction^21^, an oil droplet with lipid formation^22^, and an aqueous solution including a chemical oscillatory reaction coupled to the bromination reaction of surfactants^23^. In this study, we focus on a phenanthroline disk moving on an aqueous solution of Fe$^{2+}$ as a typical experimental system for the run-and-tumble motion of self-propelled objects (Fig. 1b and 1c).

The phenanthroline disk spontaneously and continuously swells on water without Fe$^{2+}$ in the aqueous phase (Figures S1a-i and S1b-i) due to the surface tension gradient around the disk, which originates from the surface concentration gradient of phenanthroline. The concentration gradient is caused by the coupling of the disk motion with the surface phenomena of the phenanthroline molecules, which are the supply from the disk, the diffusion of these molecules on the surface of water, and the sublimation in air^19^. Owing to the Fe$^{2+}$ present in the aqueous phase, the phenanthroline disk exhibits run-and-tumble motion (Figs. 1b, 1c, S1a-ii, and S1b-ii). The phenanthroline molecules on water are consumed by a complex reaction with Fe$^{2+}$. Thus, the driving force of the disk motion vanishes, which corresponds to the “tumble” phenomenon. During tumbling, phenanthroline is continuously supplied from the disk to water, whereas Fe$^{2+}$ is not. Thus, the concentration of Fe$^{2+}$ ([Fe$^{2+}$]) locally decreases with time due to the consumption of Fe$^{2+}$ around the disk. Then, [Fe$^{2+}$] becomes less than the threshold value for preventing disk motion. Therefore, the disk starts to move again; this motion is the “run” motion. As a result, the disk exhibits a run-and-tumble motion on the surface of an aqueous solution of Fe$^{2+}$. The typical trajectory is shown in Fig. 1b, in which the rotational diffusion of the disk is negligible.

We measured the characteristics of self-propelled motion of the phenanthroline disk on a homogeneous aqueous phase including Fe$^{2+}$. The estimated parameters were the periods of run and tumble, maximum speed of each run, and movement distance for each cycle of run and tumble. The period increased and the maximum speed decreased with an increase in [Fe$^{2+}$] (Fig. S2). Furthermore, the length of trajectory during each run was also measured, referred to as “jump length” hereafter. The jump length ($l\left( \left[ \text {Fe}^{2+}\right] \right)$) decreased with increasing [Fe$^{2+}$] (Fig. 1d). In addition, the mode of the phenanthroline disk bifurcated to uniform motion with [Fe$^{2+}$] lower than 0.3 mM. This corresponds to infinite jump length. We fitted the experimental measurements by the following logarithmic function:

1 $$\begin{aligned} l\left( \left[ \text {Fe}^{2+}\right] \right) = -3.5\ln \left( \left[ \text {Fe}^{2+}\right] - \left[ \text {Fe}^{2+}\right] _c\right) + 15.5. \end{aligned}$$

Here, [Fe$^{2+}$]$_c$ is the critical concentration for the bifurcation from intermittent oscillatory motion to uniform motion. It was estimated as 0.33 mM (see Supporting Information (SI)). The dependence of the jump length on [Fe$^{2+}$] indicates the potential of the phenanthroline disk to respond to a concentration gradient stochastically, as shown in the mathematical model of bacterial chemotaxis^5,6^.

## Bacterial chemotaxis in a self-propelled phenanthroline disk

**Figure 2:**
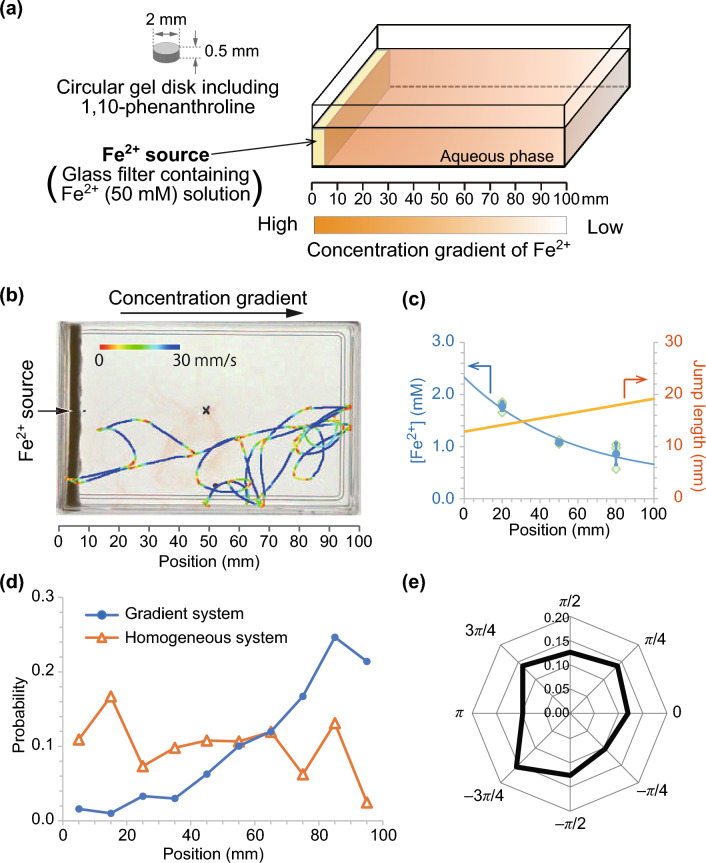
(**a**) Schematic of the experimental setup. (**b**) Trajectory of the phenanthroline disk on the [Fe$^{2+}$] gradient water phase. (**c**) Concentration gradient of Fe$^{2+}$ estimated using UV–Vis spectrometry and jump length for each position calculated by the concentration gradient and [Fe$^{2+}$] dependency of the jump length (Fig. 1d). (**d**) Existing distribution of the phenanthroline disk. The blue filled circles and orange open triangles indicate the results for the gradient water phase and homogeneous water phase ([Fe$^{2+}$] = 1.0 mM), respectively. (**e**) Probability of jump direction.

In this study, the strategy of bacterial chemotaxis was verified using a non-living self-propelled object, namely, a phenanthroline disk. A low concentration gradient of Fe$^{2+}$ was prepared in a rectangular container (see SI), where the source of Fe$^{2+}$ was placed along the left-hand edge of the container (Fig. 2a). Therefore, a concentration gradient was generated only in the *x*-direction and no gradient was present in the *y*-direction. A circular phenanthroline disk with a diameter of 2.0 mm was placed at the center of the container. It is known that the surface tension of an aqueous solution composed of simple electrolytes is almost the same as that of pure water^24^. Therefore, the surface tension was independent of the Fe$^{2+}$ concentration, which was verified by measuring the surface tension as the concentration of Fe$^{2+}$ (see SI).

The concentration gradient was experimentally estimated by measuring [Fe$^{2+}$] at three points, with the concentration values gradually decreasing from 2.0 to 0.5 mM (Figs. 2c, S3, and S4). Based on the experimental observations, the concentration gradient was fitted using the following equation:

2 $$\begin{aligned} \left[ \text {Fe}^{2+}\right] (x) = 2.0 \exp (-0.018 x) + 0.33, \end{aligned}$$

where *x* is the distance from the Fe$^{2+}$ source. By substituting this expression into Eq. (1), the jump length independent of movement direction (*l*(*x*)) can be fitted by the following linear function (Fig. 2c):

3 $$\begin{aligned} l(x) = 0.063 x + 12.9. \end{aligned}$$

As shown in the typical trajectory of a phenanthroline disk (Fig. 2b), the disk moved in both left and right directions. However, the existing distribution indicated that the disk tended to move away from the source of Fe$^{2+}$ at the left side of the container (Fig. 2d). Thus, the phenanthroline disk showed “negative chemotaxis” for the concentration gradient of Fe$^{2+}$. To clarify the effect of the chemical gradient, the existing distribution in the homogeneous aqueous phase with [Fe$^{2+}$] = 1.0 mM as a control was also observed. In this case, the existing distribution was approximately homogeneous (Fig. 2d). However, the existing probability of the disk near the edge was slightly lower than that in other regions. This may be due to the repulsive interaction will work between the particles and wall, and they are therefore less likely to stop near the wall.

The concentration gradient of Fe$^{2+}$ was estimated to be $3.6 \times 10^{-2}$ mM/mm. Therefore, the concentration difference around the disk with a diameter of 2.0 mm was $7.2 \times 10^{-2}$ mM. The surface tension of Fe(phen)$_3^{2+}$ solution was approximately constant for a concentration of Fe$^{2+}$ solution below 1 mM and only slightly decreased below 100 mM^18^. Therefore, the concentration gradient in our experiments was too small to generate a surface tension difference that would be large enough to control the direction of movement. In fact, the jump of the disk was isotropic (Fig. 2e), even though the disk was placed on the aqueous phase with a gradient of [Fe$^{2+}$]. These results indicated that the disk did not respond to the local concentration gradient of Fe$^{2+}$, but rather responded to changes in the concentration during the run, resulting in the successful realization of negative chemotaxis (Fig. 2d). These characteristics are quite similar to those of bacterial chemotaxis.

## Simple agent-based model for bacterial chemotaxis

**Figure 3:**
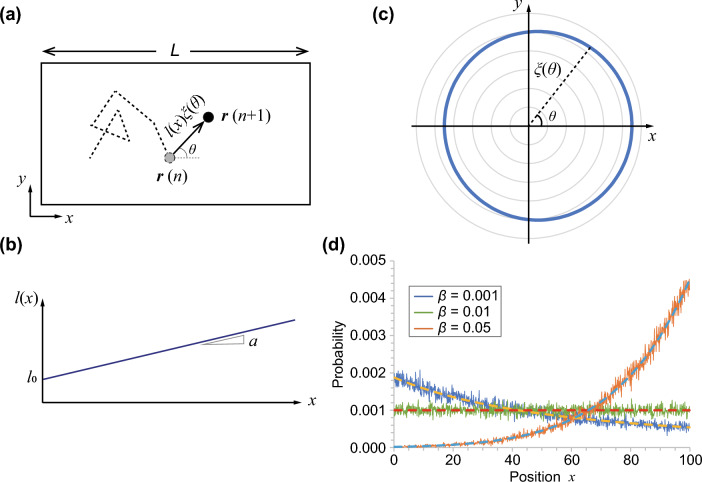
(**a**) Schematic illustration of the movement rule for a random walker. (**b**) The jump length linearly increases with the position *x*. (**c**) Anisotropic jump length depending on the movement direction ($\theta$). Here, $\xi (\theta ) = 1 + 0.1 \cos \theta$ is shown as an example. (**d**) Distribution of random walkers obtained from numerical calculation of Eq. (4), where the number of walkers (*N*) is equal to 2000. The values of the constants are $\lambda _0 = 1$, $\alpha = 0.01$, $\beta = 0.001, 0.01, 0.05$, and $L = 100$ (see SI for the relationship between the parameters in Eqs. (4) and (8)). Neumann boundary conditions were adopted. The broken lines indicate the theoretical results obtained by Eq. (10).

The phenanthroline disk stochastically responded to a low gradient of [Fe$^{2+}$] (Fig. 2d), although the disk moved isotropically on the water phase (Fig. 2e). To explain this stochastic response to a low gradient of [Fe$^{2+}$], we consider random walkers who tune their jump length. Inspired by investigations of bacterial chemotaxis, we also consider the effect of time change of concentration around the moving disk. However, our model simplified the stochastic tuning processes of the period of the run by sensing the variation in concentration over time, a method that was adopted in previously developed models^25,26^. The movement length (jump length) in our experiments was also affected by the concentration around the path of rapid motion, which is not constant in space. Thus, the concentration effect should be integrated through each path. Here, for simplicity, we assume that the jump length is determined by the concentration at the starting position of rapid motion (*x*) and movement direction against the gradient ($\theta$) (Fig. 3a). Hereafter, we refer to the former effect as the position bias and the latter as the directional bias. Furthermore, these effects are assumed to be independent of each other, even though both originate from the concentration gradient of Fe$^{2+}$ in our experiments. In our model, $\theta$ is only the random factor and the jump length is represented by the deterministic function of starting position and movement direction. It is different from the previous models using inhomogeneous Poisson processes of tumbling rate^5–8^.

4 $$\begin{aligned} {\left\{ \begin{array}{ll} \varvec{r} \left( n+1 \right) & = \varvec{r} \left( n \right) + l \left( x (n) \right) \xi \left( \theta \right) \varvec{e} \left( \theta \right) ,\\ \xi \left( \theta \right) & = 1 + b \cos \theta . \end{array}\right. } \end{aligned}$$

The functions of *l*(*x*) and $\xi (\theta )$ are the jump length depending on the position *x* and bias depending on the movement direction $\theta$, respectively, as schematically illustrated in Fig. 3b and c. $\varvec{e} \left( \theta \right)$ is the unit vector in the direction $\theta$. Here, our experiments prepare the concentration gradient only for the *x*-axis. Therefore, we assume axially symmetric for the *y*-axis in this model and consider only the projection on the *x*-axis hereafter. Thus, Eq. (4) is re-written as follows:

5 $$\begin{aligned} x \left( n+1 \right) = x \left( n \right) + l \left( x(n) \right) \left( 1 + b \cos \theta \right) \cos \theta . \end{aligned}$$

Here, to compare with the experimental observations, a linear function ($l_0 + a x$) was adopted for the function *l*(*x*). The trial number of the simulation of a single random walker (*N*), gradient (*a*), and length of the field (*L*) were fixed, whereas the value of the directional bias (*b*) was varied. Numerical calculations indicate that both positive and negative chemotaxis occur depending on the parameter *b*. The particles tended to gather at the shorter jump length region with a low value of *b*, whereas the opposite tendency was observed for a high value of *b* (Fig. 3d).

## Analytical approach

The numerically obtained bacterial chemotaxis can be explained mathematically. Our model equation (Eq. 5) is re-written as follows:

6 $$\begin{aligned} x \left( n+1 \right) = x \left( n \right) + \frac{1}{2} b l \left( x(n) \right) + l \left( x(n) \right) \left( \cos \theta + \frac{1}{2} b \cos 2 \theta \right) . \end{aligned}$$

Based on the central limit theorem, the random term $\left( \cos \theta + \frac{1}{2} b \cos 2 \theta \right)$ is replaced with Gaussian white noise with a variance $\sigma ^2$. Then, Eq. (6) is re-written as follows:

7 $$\begin{aligned} x \left( n+1 \right) = x \left( n \right) + \frac{1}{2} b l \left( x(n) \right) + l \left( x(n) \right) N \left( 0, \sigma ^2\right) , \end{aligned}$$

where $N \left( 0,\sigma ^2 \right)$ is a random variate. Based on the Itô interpretation of the stochastic integral, the following stochastic differential equation can be obtained^27–29^:

8 $$\begin{aligned} {\left\{ \begin{array}{ll} dx(t) = f(x) dt + g(x) dW,\\ f(x) = \frac{1}{2} \beta \lambda \left( x \right) , \\ g(x) = \lambda \left( x \right) \sigma , \end{array}\right. } \end{aligned}$$

where *f*(*x*) and *g*(*x*) are the drift and diffusion terms, respectively. The parameter $\beta$ and function $\lambda (x)$ are defined as $b\sqrt{\Delta t}$ and $l(x)\sqrt{\Delta t}$, respectively. Here, $\Delta t$ is the time required for one step between *n* and $n+1$. The relationship between the parameters for Eqs. (7) and (8) is discussed in the SI. Therefore, a general equation for the probability distribution of this process is given as follows^27,29^:

9 $$\begin{aligned} \frac{\partial }{\partial t} P(x,t) = -\frac{1}{2} \beta \frac{\partial }{\partial x} \left[ \lambda \left( x \right) P(x,t) \right] + \frac{1}{2} \sigma ^2 \frac{\partial ^2}{\partial x^2} \left[ \lambda \left( x \right) ^2 P(x,t) \right] . \end{aligned}$$

If we adopt a linear function of $\lambda \left( x \right) =\lambda _0 + \alpha x$, this partial differential equation can be analytically solved using the Neumann boundary condition and normalization. Using $\sigma = \frac{1}{\sqrt{2}}$, which is the standard deviation of the random function $\cos \theta$, the equilibrium distribution is expressed as follows (see SI):

10 $$\begin{aligned} P(x) = \frac{1}{c_0 } \left( \lambda _0 + \alpha x \right) ^{2(\kappa - 1)}. \end{aligned}$$

Here, $\kappa = \frac{\beta }{\alpha }$ and $c_0$ is a positive constant determined by $\alpha$, $\beta$, and field size *L*. The analytical results indicate that there is a switch between the positive and negative chemotaxis at $\kappa =1 (\alpha = \beta )$. The analytical and numerical results indicate that the use of no directional bias (i.e., $\beta$ = 0) cannot reproduce the experimental observations. As discussed below, the parameter $\beta$ was not zero and was greater than the value of $\alpha$ in our experiments. In this condition, the mathematical analysis (Eq. 10) expects that the disks tended to gather at the region with a long jump length. This agrees well with our experimental observations. In addition, it is noteworthy that our model has a potential to analyze other bacterial chemotaxis models based on inhomogeneous Poisson processes of tumbling^5–8^. In this paper, our model adopted deterministic jump length depending on both position and movement direction. However, the deterministic jump length is not necessary and can be instead of stochastic one, as long as the resulting jump length depends on the position and movement direction.

## Comparison between experimental data and mathematical analysis

**Figure 4:**
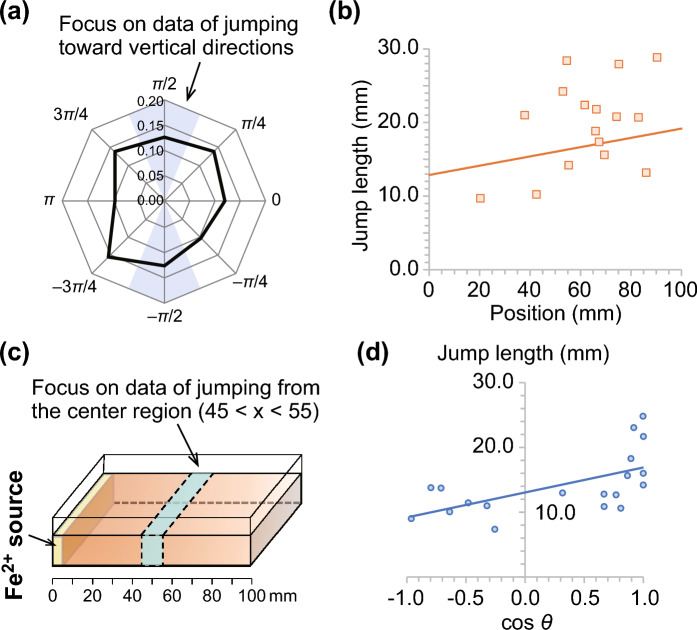
(**a**) Probability of the jump direction. (**b**) Jump lengths as a function of the position *x* of the gradient system. To eliminate the effect of the concentration gradient, the jumps toward $-\frac{5}{8}\pi \le \theta \le -\frac{3}{8}\pi$ and $\frac{3}{8}\pi \le \theta \le \frac{5}{8}\pi$, which correspond to the colored region in (**a**), were selected. The solid line is the fitting curve (Eq. 3), which is obtained by the jump length on homogeneous phase (Eq. 1) and the concentration gradient (Eq. 2). (**c**) Illustration of the focused region used to obtain the data for fitting. To eliminate the effect of the position, the data were collected only from the center ($45< x < 55$). (**d**) Jump lengths plotted against $\cos \theta$, where $\theta$ is the jump direction. The solid line indicates the linear regression line of the plotted data.

The jump length independent of the movement direction (*l*(*x*)) can be fitted using the linear function of Eq. (3) evaluated via the experimental observations considering the homogeneous aqueous phases. Here, the jump length depends on the local concentration as well as the concentration gradient. Therefore, the jump data in the direction perpendicular to the concentration gradient $\left(-\frac{5}{8}\pi \le \theta \le -\frac{3}{8}\pi , \frac{3}{8}\pi \le \theta \le \frac{5}{8}\pi \right)$ were selected and plotted against the position *x* (Fig. 4a). Although the deviations were significant, the fitting curve was well reproduced by the experimental observations (Fig. 4b). The uncertainties of parameters *a* and $l_0$ were estimated to be 0.092 and 6.0, respectively, from the plotted data.

**Figure 5:**
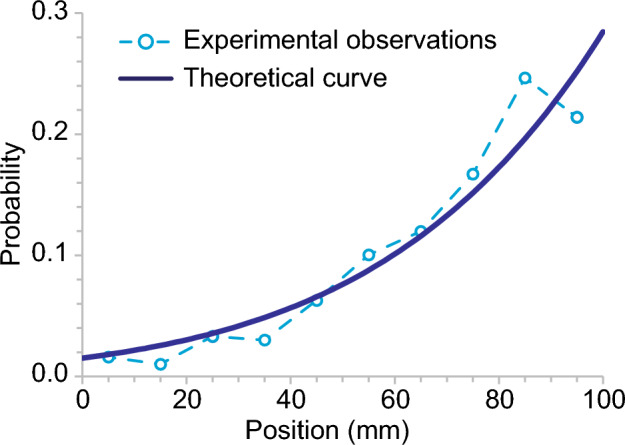
Existing distribution obtained by the theoretical equation (Eq. 10) and estimated parameters ($a = 0.092$ and $b = 0.295$). The plots of empty circle are the experimental results, which is the same as Fig. 2d.

Finally, the existing distribution of the phenanthroline disk (Fig. 2d) was compared with the expected distribution obtained by Eq. (10). As mentioned above, the parameters *a* and $l_0$ were estimated experimentally. The value of *b* can be estimated from a linear function of the jump length $|\varvec{r}(n+1) - \varvec{r}(n)|$ and $\cos \theta$, in which $\theta$ is the jump direction, even if the value of *l*(*x*) is constant. However, the value of *l*(*x*) depends on the position *x*. To eliminate this problem, we collected the jump length data starting from the center of the container, $45< x < 55$, and attempted to estimate the value of *b* (Fig. 4c). The plots were nearly a linear function of $\cos \theta$, where the fitting curve was $|\varvec{r}(n+1) - \varvec{r}(n)| = 3.85 \cos \theta + 13.1$ (Fig. 4d). Here, the maximum, second maximum, and minimum jump length data were eliminated because they highly deviated from the trend of the remaining data. The intercept of the fitting curve corresponds to *l*(*x*), and the value of *b* can be estimated as $0.295 \pm 0.125$. Therefore, the value of $\kappa$ was 4.68, namely, the effect of directional bias (*b*) was bigger than the position bias (*a*) in our experiments. Using these fitted parameters, the distribution predicted using Eq. (10) was plotted with the experimental data (Fig. 5). The fitting curve sufficiently reproduced the experimental observations. This indicates that our analytical results and parameter estimation are in good agreement with the experimental results for the bacterial chemotaxis of the phenanthroline disk.

In our experimental systems, the directional bias *b* is supposed to originate from the concentration gradient of Fe$^{2+}$. Therefore, both the directional and positional biases depend on the concentration gradient. It was also considered that the value of *b* is proportional to *a*. However, it is not yet clear if the proportionality constant, which corresponds to $\kappa$, depends on the concentration conditions or not. To clarify the relationship between the directional and positional biases, a detailed investigation based on the experimental data is required, and should be considered in future work.

## Conclusion

In this paper, we suggested a novel experiment for reproducing bacterial chemotaxis using a non-living self-propelled object. The phenanthroline disk shows a run-and-tumble motion in response to a low gradient of [Fe$^{2+}$] in the water phase, even though the jump was isotropic. Here, an isotropic jump indicates that the disk was not sensitive toward the local concentration gradient but responded to changes in [Fe$^{2+}$] during the jump. This property is similar to the behavior of bacteria. To understand the experimental results, we suggested a simple random walk model of which the jump length depends on the position and movement direction. Our simple model sufficiently reproduces the experimental observations, and the results indicate that the control of the jump length is essential in bacterial chemotaxis. In addition, the Fokker–Planck equation for our agent-based model is obtained by assuming a simplified random term. The analytically obtained steady-state solution indicates that the distribution is determined by a competition between the effects of position ($\alpha$) and movement direction ($\beta$). The mechanism is universal and can be adopted for non-living simple systems as well as more complex living organisms. Our experiments and mathematical model are for a single particle. Collective motion of such self-propelled particles is one of the challenging targets to understand fundamental mechanism of self-organization in micro-organisms.

## Experimental methods

The phenanthroline disk was composed of agar gel and phenanthroline. An agar gel sheet (thickness: 0.5 mm) was soaked in MeHO solution of phenanthroline (25 mM) for more than 12 h and washed with pure water immediately before use. The pure water was prepared by purifying with two different filters and irradiation UV light (Direct-Q UV3, Merck Millipore). After washing and drying, the phenanthroline gel sheet was cut into a circular disk with a diameter of 2.0 mm.

A water phase with an Fe$^{2+}$ concentration gradient was prepared. Pure water (66 mL) was poured into a plastic container with a width, depth, and height of 100, 65, and 28 mm, respectively. A glass filter containing 50 mM Fe$^{2+}$ aqueous solution was placed on the left side of the container. After 60 min, a phenanthroline disk was placed at the center of the aqueous phase and observed using a video camera (Handycam, Sony, 30 fps). The obtained images were analyzed using ImageJ software (NIH, USA). The disk underwent repeated resting and rapid motion, and the movement speed of the disk oscillated over time. Therefore, the jump length was estimated considering the distance it moved from the beginning to the end of this rapid motion. The threshold value of the velocity for the rapid motion was set as 10 mm s$^{-1}$.

The concentration gradient was estimated by UV–Vis spectroscopy (V-700, JASCO Corporation, Japan). The samples (200 μL of solution) were taken from the aqueous phase using a micropipette 60 min after the preparation of the aqueous phase. The sampling positions were *x* = 20, 50, and 70 mm from the Fe$^{2+}$ source. To visualize Fe$^{2+}$, the sample solutions were diluted using 1800 μL of an aqueous solution of 1,10-phenanthroline (20 mM). The concentrations of ferroin (Fe(phen)$_3^{2+}$) in the diluted solution were measured using UV–Vis spectroscopy. The measurements from the five experiments were averaged, and the concentration gradient was estimated.

## Supplementary Information

Supplementary Information 1.

Supplementary Information 2.

Supplementary Information 3.

Supplementary Information 4.

## Data Availability

All data generated or analysed during this study are included in this published article and its supplementary information files.
