# Supplementary material for: Reproduction of bacterial chemotaxis by a non-living self-propelled object
Source: Sci Rep. 2023 May 20;13:8173. doi: 10.1038/s41598-023-34788-3 (PMC10199926; doi:10.1038/s41598-023-34788-3)
Supplement: Supplementary file 2 — Supplementary Information 2. [file 41598_2023_34788_MOESM2_ESM.pdf]

Supporting Information for Reproduction of Bacterial Chemotaxis by a Non-living Self-propelled Object

Yuko Hamano[1], Kota Ikeda[2,3], Kenta Odagiri[3,4], and Nobuhiko J. Suematsu[2,3,*]

[1]School of Interdisciplinary Mathematical Sciences, Meiji University
[2]Meiji Institute for Advanced Study of Mathematical Sciences (MIMS), Meiji University
[3]Graduate School of Advanced Mathematical Sciences, Meiji University
[4]School of Network and Information, Senshu University

1 Characteristics of Self-propelled Motion on a Homogeneous Aqueous Phase

The phenanthroline disk exhibited two types of self-propelled motion: uniform motion and intermittent oscillatory motion (Fig. S1-i and S1-ii). The mode of the motion depends on the concentration of Fe^{2+} in the aqueous phase, $[Fe^{2+}]$. We examined the characteristics of the self-propelled motion of a single phenanthroline disk. The disk and homogeneous aqueous phase were prepared using the same procedure as that described in the main text. $[Fe^{2+}]$ was varied from 0.0 mM to 3.0 mM. The phenanthroline disk exhibited uniform motion in which the disk moved at a constant speed when $[Fe^{2+}]$ was low (Fig. S1-i). By contrast, the disk alternated between rapid motion and rest when $[Fe^{2+}]$ was high (Fig. S1-ii). The threshold concentration for switching from uniform motion to intermittent oscillatory motion was between 0.3 and 0.4 mM (Fig. S1c).

The period of intermittent oscillatory motion monotonically increased with $[Fe^{2+}]$ and reached a plateau at 0.8 mM (Fig. S2a). By contrast, the speed at the peak of intermittent oscillatory motion monotonically decreased

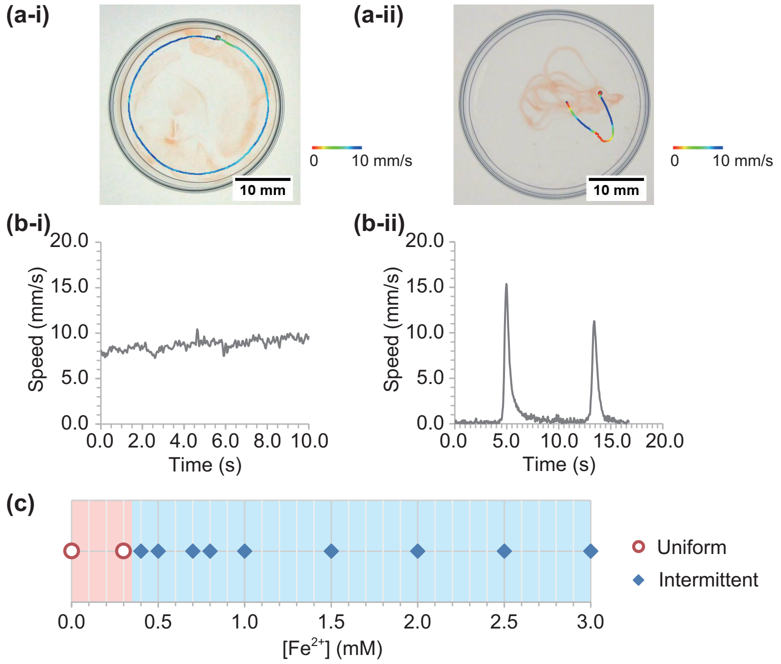

Figure S1: (a) Trajectories and (b) speed profiles of the self-propelled (i) uniform motion and (ii) intermittent oscillatory motion. The concentration of Fe^{2+} was (i) 0.3 mM and (ii) 0.8 mM, respectively. The color of the trajectory indicates the speed of motion, as indicated by the color bar. (c) Phase diagram of modes of motion. Uniform motion, indicated by red open circles, was observed up to 0.3 mM. In contrast, intermittent oscillatory motion, which corresponds to the blue-filled diamond, appeared at concentrations over 0.4 mM of Fe^{2+}.

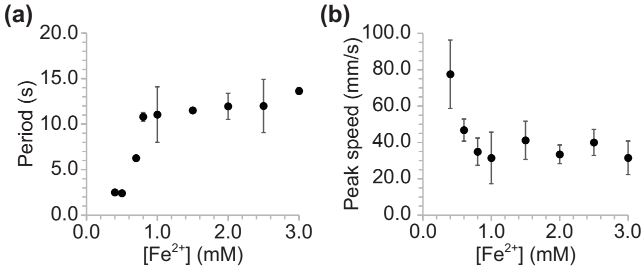

Figure S2: (a) Period and (b) peak speed of intermittent oscillatory motion depending on $[Fe^{2+}]$.

with $[Fe^{2+}]$ and approached approximately 36 mm/s at a concentration of 0.8 mM (Fig. S2b).

The jump length also depended on $[Fe^{2+}]$ in the homogeneous aqueous phase. Here, as mentioned above, the mode of the phenanthroline disk bifurcated from intermittent oscillatory motion to uniform motion when $[Fe^{2+}]$ was lower than 0.3 mM. The jump length in uniform motion corresponds to an infinite length. Therefore, the experimental measurements were fitted by the following logarithmic function:

$$l(x) = -3.5 \ln([Fe^{2+}] - [Fe^{2+}]_c) + 15.5. \tag{S1}$$

Here, $[Fe^{2+}]_c$ is the critical concentration for the bifurcation. From the fitting, it was estimated to be 0.33 mM.

2 Concentration Gradient of Fe^{2+}

A calibration curve was first obtained to estimate the concentration gradient of Fe^{2+} in an aqueous phase. An aqueous solution of $FeSO_4$ (0.01 M) was diluted with a 1,10-phenanthroline aqueous solution (4 mM). A ferroin ($Fe(phen)_3^{2+}$) solution with a concentration of 1 mM was produced. Subsequently, the ferroin solution was diluted to different concentrations (0.02 to 0.10 mM). UV-vis spectra were obtained for five solutions with different concentrations (Fig. S3a). The absorbance at the peak wavelength (510 nm) was plotted against $[Fe^{2+}]$ and fitted using a linear function (Fig. S3b). The function used for fitting is as follows:

$$Abs = 1.11 \times 10^4 [Fe^{2+}] + 0.018. \tag{S2}$$

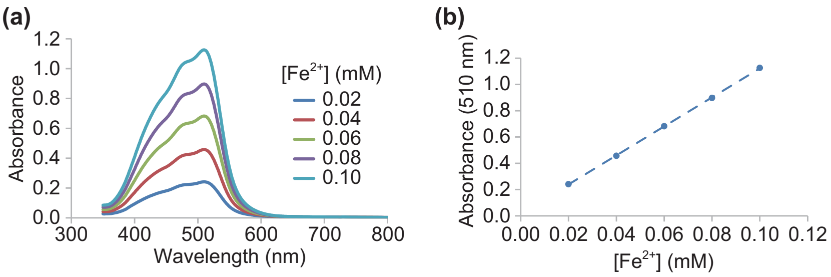

Figure S3: Calibration for $[Fe^{2+}]$. (a) UV-vis spectra for solutions with different concentrations of ferroin ($Fe(phen)_3^{2+}$). (b) Absorbance at the peak wavelength (510 nm).

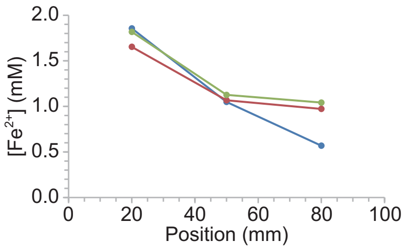

Figure S4: Concentration gradient of Fe^{2+}. Experiments were performed three times. Each estimation has been plotted with different colors.

After preparing the aqueous phase with a concentration gradient of Fe^{2+}, a negligible amount of solution (200 μL) was obtained from different positions (20, 50, and 80 mm) from the source of Fe^{2+}. Subsequently, 1800 μL of the 1,10-phenanthroline solution (20 mM) was added to each sample solution to produce a ferroin solution. The concentrations at each position were obtained using a calibration curve (Eq. S2). We performed the measurements three times, and the concentration gradient was estimated using the following equation with the average measurement of the data (Fig. S4).

$$[Fe^{2+}](x) = 2.0 \exp[-0.018x] + 0.33. \tag{S3}$$

Here, x is the position from the left edge of the container, where the source of Fe^{2+} was placed.

3 Surface Tension of Aqueous Solution of Fe^{2+}

Surface tension of aqueous solution of Fe^{2+} was measured using surface tensiometer, which used Wilhelmy method. The concentration of Fe^{2+} was changed from 0.01 mM to 50 mM. The surface tension was independent of the concentration and was almost the same value of that of pure water (Figure S5). Surface tension was measured 5 times for each concentration solution and the averaged values were plotted against the concentration of iron ion. The error bars were also plotted, but all of them were behind of the plots because of very small values.

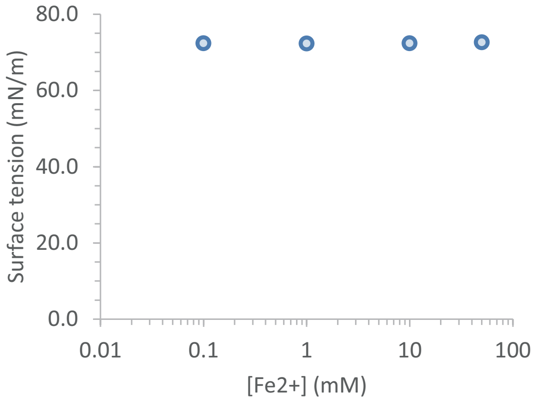

Figure S5: Surface tension of aqueous solution of Fe^{2+}. It was independent of the concentration.

4 Comparing Numerical Results using Discrete Model and Fokker-Planck Equation

The stochastic differential equation

$$dx = f(x)dt + g(x)dW \tag{S4}$$

can be expressed in the following integral form:

$$\begin{aligned} x(t+\Delta t) &= x(t) + \int_t^{t+\Delta t} f(x(s))ds + \int_t^{t+\Delta t} g(x(s))dW \\ &= x(t) + \int_t^{t+\Delta t} f(x(t))ds + \int_t^{t+\Delta t} g(x(t))dW \\ &= x(t) + f(x(t))\Delta t + g(x(t))\Delta W. \end{aligned} \tag{S5}$$

Here, $f(x)$ and $g(x)$ are assumed to be determined by $x(t)$ from the second line of the above equation. To correspond to eq. 7 in the main text, $f(x)$ and $g(x)$ should satisfy the following equations:

$$f(x)\Delta t = \tfrac{1}{2}l(x)b, \tag{S6}$$

$$g(x)\Delta W = l(x)N(0,\sigma^2). \tag{S7}$$

The ensemble average of the square of eq. S7 is

$$\begin{aligned} \left\langle |g(x)\Delta W|^2 \right\rangle &= \left\langle \left|l(x)N(0,\sigma^2)\right|^2 \right\rangle, \\ g(x)^2 \left\langle |\Delta W|^2 \right\rangle &= l(x)^2 \left\langle \left|N(0,\sigma^2)\right|^2 \right\rangle. \end{aligned} \tag{S8}$$

Due to $\left\langle |\Delta W|^2 \right\rangle = \Delta t$,

$$\begin{aligned} g(x)\sqrt{\Delta t} &= l(x)\sigma, \\ \lambda(x)\sqrt{\Delta t} &= l(x), \end{aligned} \tag{S9}$$

where $g(x)$ is defined as $\sigma\lambda(x)$. Therefore, eq. S6 should be defined as follows:

$$f(x)\Delta t = \frac{1}{2}l(x)b = \frac{1}{2}b\lambda(x)\sqrt{\Delta t}. \tag{S10}$$

To satisfy the above equation, $f(x)$ should be a constant multiple of $\lambda(x)$. Thus, we define $f(x) \equiv \frac{1}{2}\beta\lambda(x)$. Then,

$$\frac{1}{2}\beta\lambda(x)\Delta t = \frac{1}{2}b\lambda(x)\sqrt{\Delta t}. \tag{S11}$$

Finally,

$$\beta\sqrt{\Delta t} = b. \tag{S12}$$

The validity of the analytical formula of $P(x)$ (eq. S22) was verified by comparing the analytical results with the numerical results obtained using

the discrete model (eq. 4 in the main text). A comparison of eqs. 4 and 5 in the main text suggests that the parameters should satisfy the following relationship:

$$\begin{aligned} b &= \beta\sqrt{\Delta t}, \\ l(x) &= \lambda\left(x\right)\sqrt{\Delta t}, \end{aligned} \tag{S13}$$

where Δt is the time length of each step.

Here, the explicit formula of $P(x)$ (eq. S22) reveals that the numerical results should be independent of Δt. To verify this independency, we calculated six sets of parameters, which correspond to $\beta =$(a) 0.001 or (b) 0.05 and $\lambda\left(x\right) = 1 + 0.01x$ for the explicit formula $P(x)$ (eq. S22). Namely, the parameters for numerical simulation were $l_0 = \sqrt{\Delta t}$, $a = 0.01\sqrt{\Delta t}$, and $b = \beta\sqrt{\Delta t}$.

Using eq. 4 in the main text, the position x at $t = 20000$ was calculated for $N = 100000$ particles with $L = 100$. The profiles did not change for t larger than 10000. Thus, the system reached a steady state. As is shown in Fig. S6a, the profile is approximately identical for $\beta = 0.001$, independent of Δt. This is also true for $\beta = 0.05$, even when the approximation accuracy becomes low near $x = 100$ for $\Delta t = 10$. The numerical results agree well with the analytical results obtained by the Fokker-Planck equation (Fig. S6).

The existing probabilities ($P(x)$) indicated both monotonic increases and decreases depending on the value of β, as shown in Fig. S6. The phenomenon can be explained by the analytical results. As shown in eq. S22, a monotonic increase or decrease in $P(x)$ is determined by the relationship between β and α. When β is larger than α, namely, $\kappa > 1$, $P(x)$ monotonically increases. Here, α indicates the proportional constant of the jump length versus position, and β is the constant against movement direction. Therefore, the position effect is a negative taxis, and the direction is a positive taxis.

5 Derivation of Steady-state Distribution $P(x)$

Using eq. 6 in the main text, the Fokker-Planck equation can be derived as follows [1]:

$$\frac{\partial}{\partial t}P(x,t) = -\frac{\partial J}{\partial x}, \tag{S14}$$

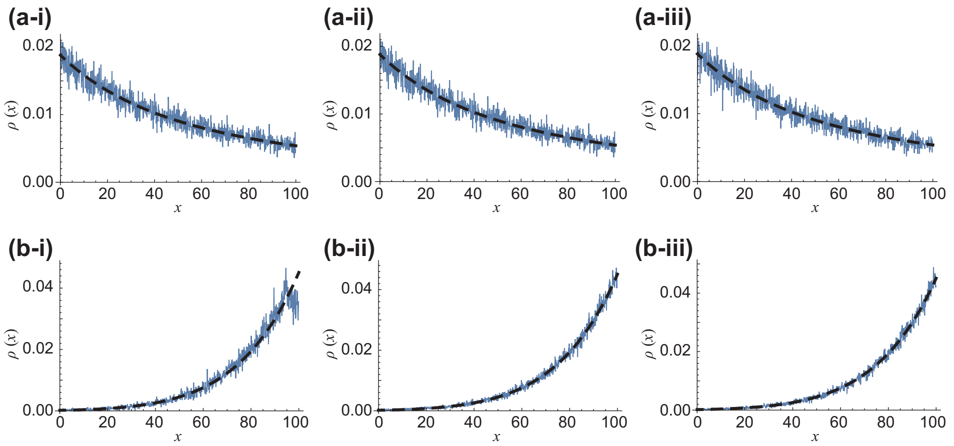

Figure S6: Numerical results obtained using discrete model (blue solid line) and analytical results obtained by Fokker-Planck equation (black broken line). The values of parameter β are (a) 0.001 and (b) 0.05. The time steps Δt for the numerical calculation are (i) 10, (ii) 0.1, and (iii) 0.001. The other parameters are $L = 100$ and $\lambda(x) = 1 + 0.01x$.

where $J = J(x,t)$ denotes the flux derived as follows:

$$J(x,t) = \left(f(x) - \frac{1}{2}\frac{\partial}{\partial x} g(x)^2 \right) P(x,t). \tag{S15}$$

We consider that a steady state of the distribution function $P(x)$, namely, eq. S14, should be 0. Thus, the following ordinary differential equation needs to be solved under the boundary conditions $J(0) = 0$ and $J(L) = 0$.

$$-\frac{d}{dx}\left(\frac{1}{2}\beta\lambda\left(x\right)P(x) \right) + \frac{\sigma^2}{2}\frac{d^2}{dx^2}\left(\lambda\left(x\right)^2 P(x) \right) = 0. \tag{S16}$$

This equation can be re-written as follows:

$$\frac{d}{dx}\left(-\frac{1}{2}\beta\lambda\left(x\right)P(x) + \frac{\sigma^2}{2}P(x)\frac{d}{dx}\left[\lambda\left(x\right)^2 \right] + \frac{\sigma^2}{2}\lambda\left(x\right)^2\frac{dP}{dx} \right) = 0. \tag{S17}$$

Therefore, $P(x)$ should satisfy the following equation:

$$\begin{aligned} &\left(-\frac{1}{2}\beta\lambda\left(x\right) + \frac{\sigma^2}{2}\frac{d}{dx}\left[\lambda\left(x\right)^2 \right] \right) P(x) + \frac{\sigma^2}{2}\lambda\left(x\right)^2\frac{dP}{dx} = 0, \\ \Rightarrow &\left[\left(-\frac{1}{2}\beta + \sigma^2\frac{d\lambda}{dx} \right) P(x) + \frac{\sigma^2}{2}\lambda\left(x\right)\frac{dP}{dx} \right] \lambda\left(x\right) = 0. \end{aligned} \tag{S18}$$

Therefore, the problem is

$$\lambda\left(x\right)\frac{dP}{dx} = \left(\frac{1}{\sigma^2}\beta - 2\frac{d\lambda}{dx}\right)P(x). \tag{S19}$$

Thus, the steady state of the distribution can be described as follows:

$$P(x) = \frac{1}{c_0\lambda\left(x\right)^2}\exp\left[\int_0^x \frac{\beta\sigma^{-2}}{\lambda\left(y\right)}dy\right], \tag{S20}$$

where c_0 is a positive constant defined as $P(x)$ and satisfies the following normalizing condition:

$$\int_0^L P(x)dx = 1. \tag{S21}$$

To obtain the explicit formula for eq. S20, we consider the linear function $\lambda\left(x\right) = \alpha x + \lambda_0$. In addition, standard deviation σ is set to $\frac{1}{\sqrt{2}}$, which is equal to the standard deviation of random function $\cos\theta$. Using these functions, the steady-state function of $P(x)$ can be derived as follows:

$$\begin{aligned} P(x) &= \frac{1}{c_0\lambda_0^{2\kappa}}\left(\lambda_0 + \alpha x\right)^{2(\kappa-1)}, \\ \kappa &= \frac{\beta}{\alpha}, \\ c_0 &= \begin{cases} \dfrac{1}{\alpha\lambda_0\left(2\kappa-1\right)}\left[\left(1+\dfrac{\alpha L}{\lambda_0}\right)^{2\kappa-1} - 1\right] \quad (\kappa \neq \frac{1}{2}), \\ \dfrac{1}{\alpha\lambda_0}\ln\left(1+\dfrac{\alpha L_0}{\lambda_0}\right) \quad (\kappa = \frac{1}{2}). \end{cases} \end{aligned} \tag{S22}$$

6 Phenomenological consideration of origin of the directional bias b

The directional bias b would originat from the concentration gradient of $Fe_2{}^{+}$, based on the following phenomenological consideration. The driving force of self-propelled motion is induced by the surface activity of phenanthroline. Thus, the concentration of $Fe_2{}^{+}$ around disk is high enough, in which the phenanthroline is consumed rapidly, and thus, the disk cannot obtain enough driving force. This is rest state. During rest state, $Fe_2{}^{+}$ is also consumed and the concentration decreased around the resting disk, and then, the disk starts to move when $[Fe_2{}^{+}]$ becomes lower than the threshold value. In this time, low $[Fe_2{}^{+}]$ reagion was formed by consumption reaction

and molecular diffusion. By briefly consideration, the movement distance might be determined by the size of the low concentration region, because $[Fe_2{}^+]$ is high enough to prevent movement at the out of the region.

With assuming that the low concentration region was determined by the diffusion of phenanthroline, the amount of consumption can be estimated as follows [**?**]:

$$
\begin{aligned}
[\mathrm{Phen}](t) &= c_0(t) \exp\left[-\frac{(x-x_0)^2}{4Dt}\right] - \frac{s_0(x-x_0)}{2D}\left(\mathrm{sgn}(x-x_0) - \mathrm{erf}\left(\frac{x-x_0}{\sqrt{4Dt}}\right)\right), \\
c_0(t) &= s_0\sqrt{\frac{t}{\pi D}},
\end{aligned}
\tag{S23}
$$

where, x_0 is the starting position of disk, D is diffusion constant, s_0 is supply rate. Due to supply from the disk, the formation is different from usual diffusion formula.

References

[1] H. Risken, "The Fokker-Planck equation: Methods of solution and applications" Springer Series in Synergetics, 18, 2nd ed. (1996).
